# The electrophysiological effects of Tongyang Huoxue granules on the ignition phase during hypoxia/reoxygenation injury in sinoatrial node cells

**DOI:** 10.3389/fphys.2024.1402478

**Published:** 2024-06-07

**Authors:** Qiaomin Wu, Xing Chang, Yanli Wang, Jinfeng Liu, Xuanke Guan, Zhiming Liu, Ruxiu Liu

**Affiliations:** Guang’ Anmen Hospital, China Academy of Chinese Medical Sciences, Beijing, China

**Keywords:** Tongyang Huoxue granules, sinus node dysfunction, ignition phase, INCX, ICaL

## Abstract

**Introduction:**

This study was undertaken to explore the potential therapeutic effects of Tongyang Huoxue Granules (TYHX) on sinoatrial node (SAN) dysfunction, a cardiac disorder characterized by impaired impulse generation or conduction. The research question addressed whether TYHX could positively influence SAN ion channel function, specifically targeting the sodium-calcium exchanger (*I*_NCX_) and L-type calcium channel (*I*_CaL_) of the SAN.

**Methods:**

Sinoatrial node cells (SANCs) were isolated and cultured from neonatal Japanese big-eared white rabbits within 24 h of birth. The study encompassed five groups: Control, H/R (hypoxia/reoxygenation), H/R+100 μg/mL TYHX, H/R+200 μg/mL TYHX, and H/R+400 μg/mL TYHX. The H/R model, simulating hypoxia/reoxygenation stress, was induced within 5 days of culture. Whole-cell patch clamp technique was employed to record currents following a 3-min perfusion and stabilization period with TYHX.

**Results:**

TYHX administration demonstrated improvements in the ignition phase of impaired SANCs. The half-maximal effective dose of TYHX, as determined by SANC beating frequency, was found to be 323.63 μg/mL. Inward current density of *I*_NCX_ increased in response to TYHX (200 and 400 μg/mL), while TYHX enhanced *I*_CaL_ current density in H/R SANCs, with 400 μg/mL exhibiting greater efficacy. Additionally, TYHX regulated the gating mechanisms of *I*_CaL_ by right-shifting the steady-state inactivation curve and accelerating recovery from inactivation. Notably, TYHX increased the activation time constant under 200 and 400 μg/mL, prolonged the fast inactivation time constant τ1 with 400 μg/mL, and extended the slow inactivation time constant τ2 with 100 and 400 μg/mL.

**Discussion and conclusion:**

The findings suggest that TYHX may hold promise as a therapeutic intervention for sinus node dysfunction, offering potential avenues for drug development aimed at safeguarding SAN function.

## 1 Introduction

Sick sinus syndrome (SSS) is a condition in which the sinus node or peripheral tissue is damaged, leading to various arrhythmias caused by impaired conduction of sinus node pacing impulses. Electrocardiographic manifestations of SSS include sinus bradycardia, sinus arrest, sinoatrial block, or bradycardia-tachycardia syndrome. Sick sinus syndrome is commonly found in middle-aged and elderly individuals, with an estimated prevalence of approximately 1 case per 600 adults aged 65 and above (Dobrzynski et al., 2007). As age increases, the incidence of SSS also rises, and it is expected to increase sharply in the next 50 years (Jensen et al., 2014). Pharmacological treatment for sinus node dysfunction has limitations. Western medicines such as atropine and adrenaline are typically used for acute sinus node dysfunction, but there are currently no effective drug treatments for chronic sinus node dysfunction. However, several studies have found that natural drugs can protect the sinus node and regulate the pacing mechanism. Therefore, developing traditional Chinese medicine may have significant implications for the treatment of chronic SSS.

The pathogenesis of SSS lies in the disruption of the “calcium clock” and “membrane clock” in sinoatrial node cells (SANC), which involves the imbalance of sarcoplasmic reticulum calcium handling, calcium release, and alterations in ion channels on the cell membrane. In which, the funny current (*I*_f_) plays a pivotal role in the initial phase 4, facilitating a gradual increase in cell membrane potential. Subsequently, the T-type calcium current and L-type calcium current activate sequentially, further driving membrane potential depolarization. Concurrently, the opening of RyR_2_ receptors in the sarcoplasmic reticulum creates the necessary conditions for phase 0 depolarization. As intracellular calcium ion concentration continues to rise, this change triggers the sodium-calcium exchanger, fostering futher cell depolarization.

In recent years, studies have found that there is a positive feedback mechanism in the clock coupling stage of SANC, which plays a role in accelerating diastolic depolarization (DD) to the action potential (AP) threshold potential, known as the “ignition phase.” In 2018, Lyashkov et al. (2018) analyzed APs using the time derivative of membrane potential (dV/dt) and identified the “ignition phase” as a critical stage in pacemaker generation. Their research showed that the rate of feed-forward crosstalk during the “ignition” phase rapidly accelerates to 0.15 V/s, marking the beginning of the “ignition phase” and the time when the inward amplitude of *I*_NCX_ increases during DD. When dV/dt reaches 0.5V/s, it indicates that depolarization has reached the critical membrane potential for generating an AP (Lyashkov et al., 2018), which is also the moment when *I*_CaL_ rapidly increases. There is also a linear correlation between the time to ignition phase and the AP cycle length using human SANC (Lyashkov et al., 2018), which means that the ignition stage is closely related to regulating SANC AP rhythm. Studies have indicated that the NCX channel protein is expressed in the sinoatrial node (SAN) and ranks among the top 16% of proteins in this region (Linscheid et al., 2019). Calcium channels and their subordinate subunits also exhibit higher expression levels in the sinoatrial node than in the atrium (Linscheid et al., 2019). Thus, the ignition phase involved by *I*_NCX_ and *I*_CaL_ is a key pacing mechanism for spontaneous AP generation in the sinus node.

Tongyang Huoxue Granule (TYHX) was developed by Professor Liu Zhiming, a master of traditional Chinese medicine. This formula contains *Astragalus membranaceus*, *Aconitum carmichaeli* Debx., *Panax ginseng* C. A. Mey., *Polygonatum sibiricum* Redouté, and *Panaxnotoginseng* (Burk.) F.H.Chen. According to contemporary pharmacological studies, those traditional Chinese medicines are rich in flavonoids, polysaccharides, and alkaloids, including astragaloside IV, demethyl aconitine, ginsenosides, and polygonatum polysaccharides. These compounds contribute to antioxidative effects, scavenging of free radicals, improved heart contraction, and bolstered immune function (Zhang et al., 2021). In clinical research, we have confirmed that TYHX can increase patients’ average heart rate by a median of 5.19 beats per minute and improve their quality of life as assessed by the SF-36. Basic research has found that TYHX accelerates the activation and decreases the inactivation of ultrarapid delayed rectifier potassium current (*I*_kur_) and transient outward potassium current (*I*_to_), shortening the 3-phase repolarization time of AP (Wang et al., 2022). Additionally, TYHX has multiple effects, including inhibiting calcium overload, restoring mitochondrial membrane potential levels, regulating mitochondrial fusion/fission, improving mitochondrial autophagy levels, and maintaining mitochondrial energy metabolism (Chang et al., 2023). However, the transition process of TYHX on AP from late DD to phase 0 and the current changes mediated by the ignition phase are still unclear. In this study, we aim to determine the protective effect of TYHX on cellular electrophysiology of the ignition phase using the hypoxia/reoxygenation (H/R) model of neonatal rabbit SANC.

## 2 Material and methods

### 2.1 Reagents

Tongyang Huoxue Granules (*A. membranaceus*, *A. carmichaeli* Debx., *P. ginseng* C. A. Mey., *Polygonatum sibiricum* Redouté, and *Panaxnotoginseng* (Burk.) F.H.Chen) were provided by Jiangyin Pharmaceutical Co., Ltd (batch number: 1811312), were prepared by decoction and alcohol extraction. Ultrapure water was used to dissolve TYHX (1 g/mL), and adjusted the pH value to 7.4-7.6. Then sterilized TYHX solution using 0.22 μm microporous membrane filter.

The internal pipette solution of AP contained (in mM) K-aspartate 120, KCl 20, MgCl_2_·6H_2_O 1, Na_2_ATP·3H_2_O 4, HEPES 10, and Glucose 10 (pH 7.2, adjusted with NaOH). The bath solution of AP contained (in mM) NaCl 140, CaCl_2_ 1, MgCl_2_·6H_2_O 1, HEPES 10, Glucose 5 and KCl 4 (pH 7.4, adjusted with NaOH).

The internal pipette solution of *I*_NCX_ contained (in mM) CsCl 100, TEACl 20, NaCl 10, MgCl_2_·6H_2_O 0.5, Na_2_ATP·3H_2_O 5, and HEPES 10 (pH 7.2, adjusted with CsOH). The bath solution of *I*_NCX_ contained (in mM) NaCl 140, CaCl_2_ 2, MgCl_2_·6H_2_O 2, HEPES 5, Glucose 10, BaCl 1, CsCl 2, nifedipine 0.02 and Ouabain 0.02 (pH 7.4, adjusted with NaOH).

The internal pipette solution of *I*_CaL_ contained (in mM) CsCl 120, CaCl_2_ 1, MgCl_2_·6H_2_O 5, HEPES 10, EGTA 11 and Na_2_ATP·3H_2_O 5 (pH 7.2, adjusted with CsOH). The bath solution of *I*_CaL_ contained (in mM) NaCl 140, CaCl_2_ 2, MgCl_2_·6H_2_O 1, HEPES 10, Glucose 10 and KCl 4 (pH 7.4, adjusted with CsOH).

The hypoxia solution contained (in mM) NaHCO_3_ 6, KCl 10, NaH_2_PO_4_ 0.9, MgSO_4_ 1.2, NaCl 98.5, CaCl_2_ 1.8, HEPES 20 and Sodium lactate 40 (pH 6.8, adjusted with HCl). The reoxygenation solution contained (in mM) NaH_2_PO_4_ 0.9, KCl 5, NaCl 129.5, NaHCO_3_ 20, MgSO_4_ 1.2, CaCl_2_ 1.8, HEPES 20, Glucose 55% and 10% FBS (pH 7.4, adjusted with NaOH).

### 2.2 Isolation and culture of neonatal rabbit SANCs

Neonatal rabbit SANCs were isolated and cultured according to previously published methods (Chang et al., 2023). Newborn Japanese big-eared white rabbits (within 24 h) were obtained from Beijing Long’an Experimental Animal Breeding Center (production license: SCXK (Beijing) 2019-0006). The animal experiments were conducted in accordance with a protocol that received approval from the Institutional Animal Research Committee. 4-6 neonatal rabbits were selected for each experiment. After anesthesia with isoflurane, the neonatal rabbits were fixed on an operating table, and the chest was disinfected with 75% alcohol. The heart was exposed, and a 2 mm^3^ piece of tissue directly below the right auricle and between the superior vena cava was excised and placed in DMEM (without FBS) pre-cooled to 4 °C. The tissue was washed 2-3 times with pre-cooled PBS and minced into small pieces (0.3 mm^3^). The tissue fragments were then incubated with 8 mL of 0.08% trypsin (Sigma, United States) in a 37°C water bath for 5 min. The suspension was gently agitated for 1 min, allowed to settle for 30 s, and the supernatant was discarded. The tissue fragments were then incubated with 5 mL of 0.1% type II collagenase (Worthington, United States) in a 37°C water bath and gently agitated for 5 min. The suspension was again gently agitated for 1 min, allowed to settle for 30 s, and the supernatant was collected and placed in a 50 mL centrifuge tube containing complete DMEM with 20 mL of 10% FBS. The resulting cell suspension was filtered through a 100 μm cell strainer (Corning^®^) and centrifuged at 900 r/min for 5 min. The supernatant was discarded, and the cells were resuspended in complete DMEM. The single-cell suspension was inoculated onto cell culture dishes and incubated at 37°C in a 5% CO_2_ incubator for 90 min. The differential adherent method was then used to separate fibroblasts. The culture medium was changed after 24 h, and subsequently changed every other day. When changing the medium, 5-bromouridine (BrU) was added at a final concentration of 0.1 mM to inhibit fibroblast growth. Cells from each group were isolated from 5-8 rabbits.

### 2.3 Establishment of hypoxia/reoxygenation model in SNCs

The hypoxia/reoxygenation model was produced using a method previously reported (Wang et al., 2022). The original culture medium was replaced with a pre-N2-saturated hypoxia solution, and the cells were cultured in a cell hypoxia incubator with 95% N_2_ and 5% CO_2_ for 1 h. After 1 h, the hypoxia solution was removed, and a reoxygenation solution was added to restore oxygen and sugar supply to the cells. The cells were then cultured in a 5% CO_2_ cell incubator for 3 h.

### 2.4 Electrophysiological recordings

We used a standard whole-cell patch clamp to record currents under previously described conditions (Wang et al., 2022). Signals were collected and analyzed using Axopatch 700B amplifier (Axon Instruments, United States) and pCLAMP 10.4 software (Axon Instruments, United States). The resistance of the glass microelectrode was set at 4–6 MΩ. TYHX was administered through acute perfusion. The cells were stabilized for 3 min after perfusion, and the recording was completed within 30 min.

To record the spontaneous action potential (AP), the recording mode was switched to current clamp. We recorded the spontaneous action potential of SANC at the cell’s resting membrane potential without any frequency stimulation. Select cells exhibiting the spontaneous AP characteristic of the sinoatrial node, switch to voltage clamp mode, and record *I*_NCX_ and *I*_CaL_. To record *I*_NCX_, the cell clamp was set at −60mV, and the voltage was then depolarized from 80 mV to −120 mV in a ramp voltage pulse manner at a speed of 90 V/s and recovered to −60 mV. *I*_NCX_ current was calculated as the difference of current before and after exposure to NiCl_2_ (5 mM). To elicit *I*_CaL_, we applied step depolarizations from −40 mV to +50 mV in 10 mV increments, each with a 200 ms pulse-width, while holding the potential at −80 mV. The stimulation frequency was set at 0.1 Hz. We also induced a steady-state activation (SSA) curve using the same voltage steps. The SSA curve was fitted to the Boltzmann equation: g/g_max_ = 1/(1+exp[(*V*_1/2,act_-*V*_m_)/k_act_]), where g_max_ is the maximum slope conductance, *V*_m_ is the test potential, *V*_1/2,act_ is the half-maximal activation voltage and k_act_ is the slope factor. The steady-state inactivation (SSI) curve was recorded through the two-pulse impulse stimulation method. We applied voltage steps between −40 and +60 mV, with 10 mV steps and 1000 ms pulse width, and then a 200 ms, 0 mV test pulse. The SSI curve was fitted to the Boltzmann equation: *I*/*I*_max_ = 1/(1+exp[(*V*_1/2,inact_-*V*_m_)/k_inact_]), where *V*_1/2,inact_ is the voltage of half inactivation and k_inact_ is the slope factor. Activation and inactivation kinetics of *I*_CaL_ were recorded using a single stimulus approach. The activation kinetics of *I*_CaL_ were fitted by a single-term exponential function: *I*(t) = A_0_(1-exp(1-t/τ)) (A_0_ is the steady-state current amplitude, t is the point of active phase time, τ is the time constant of activation). Inactivation kinetics of *I*_CaL_ was fitted by the binomial exponential equation: *I*(t) = A_0_+A_1_(1-exp(1-t/τ_1_))+A_2_(1-exp(1-t/τ_2_)) (τ_1_ is the fast inactivation time constants, τ_2_ is the slow inactivation time constants). Recovery from the inactivation curve (RFI) was constructed at a potential of −40 mV, applying 150 ms and 0 mV square wave stimulation, and then repolarizing to −40 mV. Subsequently, a second 150 ms and 0 mV square wave stimulation was applied at intervals of 5, 10, 20, 40, 80, 160, 320, 640, 1280, 2560, and 5120 ms, respectively. The RFI curve also was fitted by binomial exponential equation, where τ_1_ is the fast recovery time constant, τ_2_ is the slow recovery time constant. No P/N leak subtraction was applied in the methodology.

### 2.5 Data statistics and analysis

Origin 2018, Graph Pad Prism 8.0 software, and SPSS 20.0 software are used for graphic and statistical analyses. Measurement data were presented as mean ± standard error of mean (SEM). ANOVA analysis was used for intergroup comparison that conformed to normal distribution and homogeneity of variance. Kruskal Wallis H-test analysis was used for intergroup comparison with non-normal distribution or unequal variance. Results were considered statistically significant for *p* < 0.05 and highly significant for *p* < 0.01.

## 3 Results

### 3.1 Effects of TYHX on the spontaneous AP and ignition phase of the H/R SANCs

**FIGURE 1:**
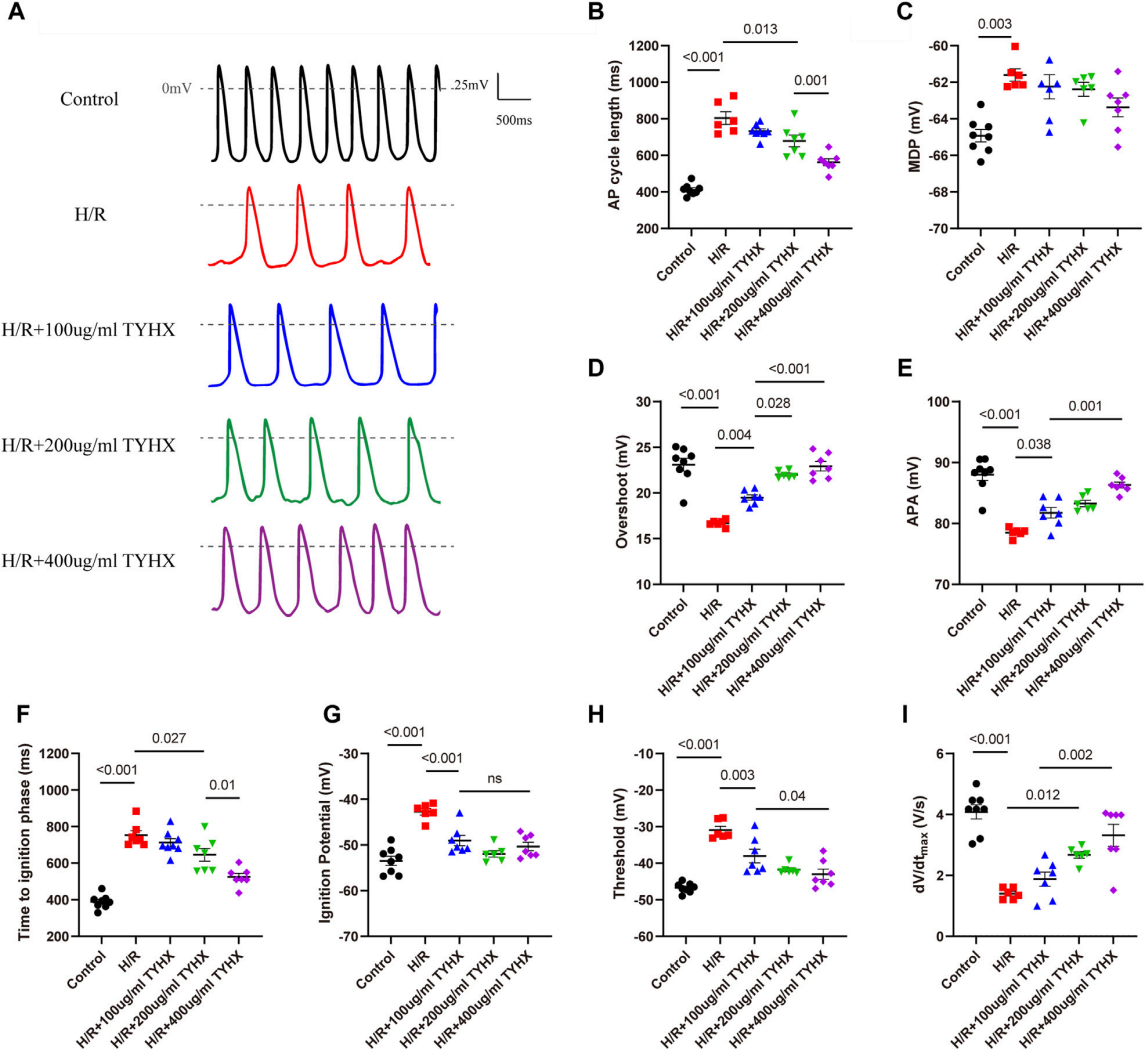
Effects of TYHX on the spontaneous action potentials of the H/R SANCs. **(A)** Spontaneous action potential current traces. **(B)** Cycle length of AP in sinoatrial node cells (*n* = 6–8). **(C)** maximal diastolic potential of AP in sinoatrial node cells (*n* = 6–8). **(D)** Overshoot of AP in sinoatrial node cells (*n* = 6–8). **(E)** Action potential amplitude in sinoatrial node cells (*n* = 6–8). **(F)** Time to ignition phase of sinoatrial node cells. **(G)** Ignition potential in sinoatrial node cells (*n* = 6–8). **(H)** Threshold potential in sinoatrial node cells (*n* = 6–8). **(I)** maximum time derivative of AP membrane potential in sinoatrial node cells (*n* = 6–8).

We first evaluated the effects of TYHX on the automaticity of the H/R SANCs. We found spontaneous AP (SAP) was decreased, and CL was longer, from 412.1 ± 11.01 ms to 804 ± 42.63 ms during hypoxia-reoxygenation (Figures 1A, B). In order to determine if there were other changes to the action potentials, we also measured maximal diastolic potential (MDP), overshoot (OS) and AP amplitude (APA) (Figures 1C–E). The results showed that the H/R injury depolarized the MDP, decreased the OS and APA (MDP: H/R: 61.61 ± 0.34 vs. Ctrl: 64.93 ± 0.34 mV, n = 6–8, *p* < 0.01; OS: H/R:16.69 ± 0.15 vs. Ctrl: 23.09 ± 0.69 mV, n = 6–8, *p* < 0.01; APA: H/R:78.48 ± 0.30 vs. Ctrl:88.02 ± 0.96 mV, n = 6–8, *p* < 0.01). In the TYHX groups, it was found that 200 and 400 μg/mL shortened the CL (200 μg/mL: 679.1 ± 31.76 ms, 400 μg/mL: 562.4 ± 18.82 ms, n = 7, *p* < 0.05), which significantly increased the SAP. TYHX also improved the OS and APA at different concentrations (OS: 100 μg/mL: 19.5 ± 0.29 mV, 200 μg/mL: 22.02 ± 0.21 mV, 400 μg/mL: 22.93 ± 0.52 mV, n = 7, *p* < 0.01; APA: 100 μg/mL: 81.75 ± 0.87 mV, 200 μg/mL: 83.27 ± 0.65 mV, 400 μg/mL: 86.3 ± 0.47 mV, n = 7, *p* < 0.01). However, TYHX had little effect on the MDP.

The above experimental results suggest that TYHX can improve the automaticity of impaired SANCs, but the specific mechanism needs further investigation. Based on recent theoretical and experimental studies, it has been shown that ignition onset occurs when the time derivative of the AP membrane potential (dV/dt) is equal to 0.15 V/s, and reaches take-off potential when dV/dt = 0.5 V/s, which is considered the AP threshold(Lyashkov et al., 2018). Thus, we further analyzed changes in ignition phase parameters, including time to ignition phase (TIP), ignition potential (IP), and threshold potential (TP), and calculated the time derivative of the AP membrane potential (dV/dt) of the sinoatrial node cells.

In the H/R group, we found that the injury prolonged TIP and depolarized the IP and TP (Figures 1F–H). Furthermore, the dV/dt_max_ slowed significantly in response to H/R (Figure 1I). In the TYHX groups, the results showed that 200 and 400 μg/mL of TYHX could reduce TIP and accelerate dV/dt_max_. Hyperpolarized IP and TP were found in all TYHX groups. Specifically, there was no significant difference in TP and dV/dtmax between the 100 and 200 μg/mL TYHX concentrations (*p* > 0.05). However, the 400 μg/mL TYHX concentration showed significant differences in TP and dV/dtmax, with *p* values less than 0.05 and 0.01, respectively, compared to the 100 μg/mL TYHX concentration.

These results suggested that hypoxia/reoxygenation injury leads to a slower speed of AP and delayed ignition phase, while TYHX promotes ignition occurrence. Furthermore, based on the observed impact of TYHX on enhancing the beating frequency of SANC, it was found that the half-maximal effective dose of TYHX is 323.63 μg/mL.

### 3.2 Effect of TYHX on *I*_NCX_ in H/R SANCs

During the ignition phase, the rhythmic release of calcium generated by the sarcoplasmic reticulum (SR) stimulates the Na/Ca^2+^ exchanger (NCX) channel and rapidly depolarizing the cell membrane by activating the *I*_CaL_, promoting the AP to reach TP level. *I*_NCX_ links the “calcium clock” and “membrane clock” and the onset of the ignition phase. To get further insights into ignition onset, we analyzed the *I*_NCX_.

**FIGURE 2:**
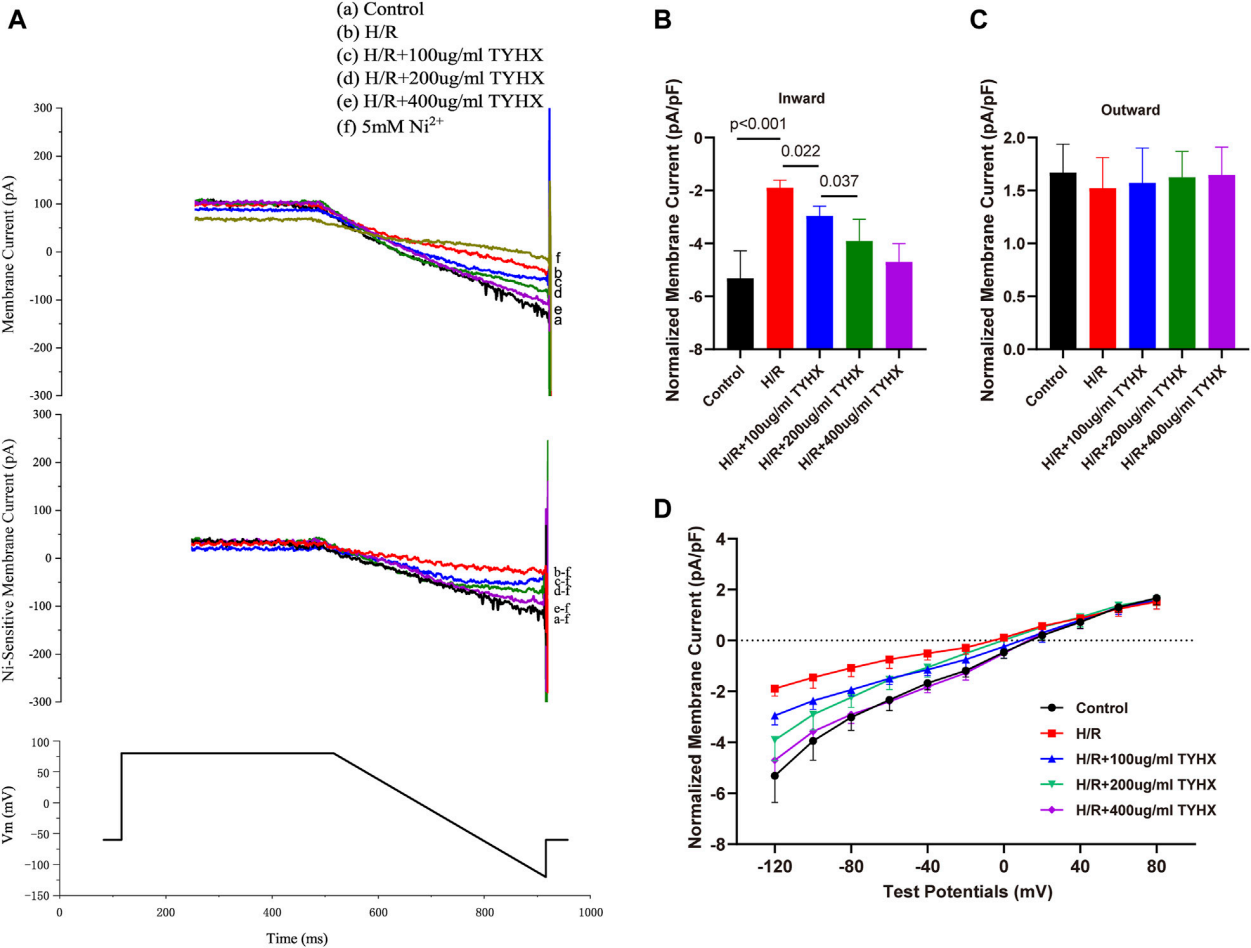
Effect of TYHX on *I*_NCX_ in H/R SANCs. **(A)** Representative *I*_NCX_ traces were recorded from isolated neonatal rabbits’ SAN cells in five groups. **(B)** Maximum inward current density of *I*_NCX_ (*n* = 6–10). **(C)** Maximum outward current density of *I*_NCX_ (*n* = 6–10). **(D)** I-V curve of *I*_NCX_ (*n* = 6–10).

Compared with the control group, the amplitude of the inward current in the H/R group decreased; in the TYHX groups, the inward current amplitude of *I*_NCX_ gradually increased (Figure 2A). It could be found that the maximum inward current density of *I*_NCX_ in the H/R group decreased significantly from −5.32 ± 0.43 pA/pF to −1.90 ± 0.11 pA/pF (n = 6–7, *p* < 0.05) (Figure 2B). The maximum inward current density was increased in response to TYHX at different concentrations (100 μg/mL: 2.96 ± 0.11 pA/pF, 200 μg/mL: 3.91 ± 0.29 pA/pF, 400 μg/mL: 4.71 ± 0.26 pA/pF, n = 7–10, *p* < 0.05). Compared with the TYHX-100 μg/mL group, the 200 and 400 μg/mL group increased more maximum inward current density of *I*_NCX_ (*p* < 0.05). However, it seems to be little difference in maximum outward current density between groups (Figure 2C).

We also analyzed the voltage dependence of *I*_NCX_. The current-voltage (I-V) curve results show that the *I*_NCX_ current has a reversal potential between −20 and 20 mV. Compared with the Control group, the inward current of *I*_NCX_ in the H/R group decreased from 0 mV to 120 mV (*p* < 0.05) (Figure 2D). After TYHX intervention, the inward current density increased within the range of −20 mV to −120 mV (*p* < 0.05). With the increase in drug concentration, the I-V curve increased more significantly. These results indicate that TYHX primarily affects the forward transport mode of *I*_NCX_ in a voltage-dependent manner.

### 3.3 Effect of TYHX on the current density and I-V curves of *I*_CaL_ in H/R SANCs

**FIGURE 3:**
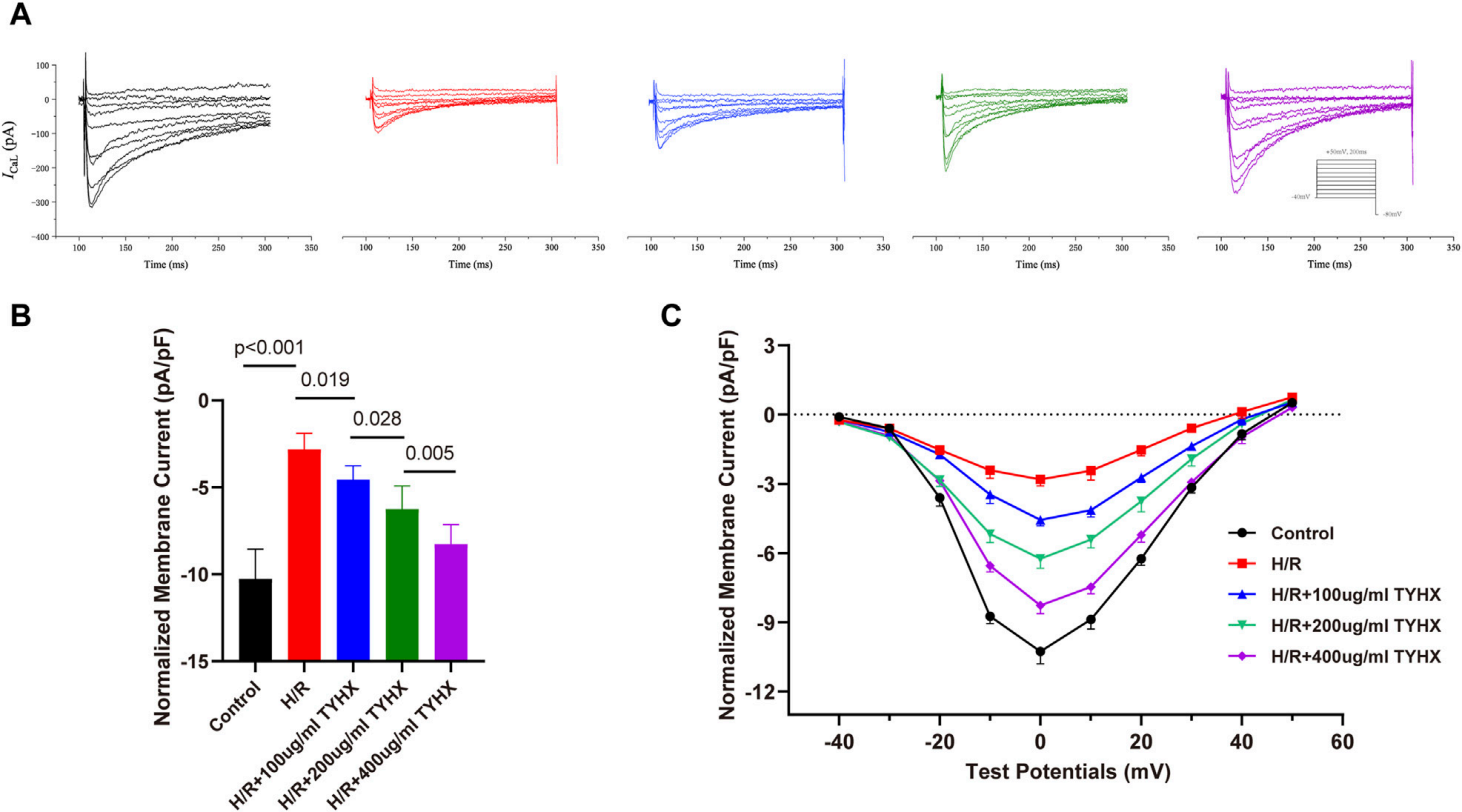
Effect of TYHX on the current density and I-V Curves of *I*_CaL_ in H/R SANCs. **(A)** Representative *I*_CaL_ traces were recorded from isolated neonatal SANCs in five groups. **(B)** Peak current density of *I*_CaL_ (*n* = 10). **(C)** I-V Curves of *I*_CaL_ in neonatal SANCs (*n* = 10).

After the ignition phase occurs, activated *I*_CaL_ promote the DD to reach the AP threshold, and its mediated Ca^2+^ influx enhances calcium release from SR, which constructs positive feedback. As widely recognized, hypoxia diminishes the activity of calcium channels in coronary artery muscle cells (Calderón-Sánchez et al., 2009) and cardiac myocytes(Hool and Arthur, 2002), a phenomenon linked to oxidative stress damage induced by hypoxia (Kiselyov and Muallem, 2016). Prior research indicates that the TYHX can ameliorate various pathological injuries in sinoatrial node cells under hypoxic conditions(Chang et al., 2023). Thus, we explored changes in *I*_CaL_ after the intervention of H/R and TYHX under voltage clamp conditions in SANCs. The *I*_CaL_ current graph shows that compared to the Control group, the *I*_CaL_ amplitude of the H/R group was decreased. In the TYHX groups, the current amplitude was increased with the increase of the concentration of TYHX (Figure 3A). We further analyzed the peak current density of *I*_CaL_ (Figure 3B): Compared with the Control group (−10.26 ± 0.54 pA/pF, n = 10), the peak current density in the H/R group was significantly lower (−2.80 ± 0.29 pA/pF, n = 10, *p* < 0.01). Different concentrations of TYHX could increase the peak current density (100 μg/mL: 4.56 ± 0.27 pA/pF, 200 μg/mL: 6.24 ± 0.42 pA/pF, 400 μg/mL: 8.27 ± 0.36 pA/pF, n = 10, *p* < 0.05). Compared to the 100 μg/mL TYHX group, the 200 μg/mL and 400 μg/mL TYHX groups exhibited a greater number of statistically significant differences (*p* < 0.05). The aforementioned results suggest that TYHX partially regulated *I*_CaL_ under hypoxia/reoxygenation injury.

The *I*_CaL_ I-V curve presents an inverted bell shape, reaching its maximum at 0 mV. Compared with the Control group, the *I*_CaL_ current density in the H/R group decreased significantly between −20 mV and 30 mV (*p* < 0.05), but the I-V curve shape remained unchanged. The *I*_CaL_ current density increased from −10 mV to 30 mV in response to TYHX with different concentrations (*p* < 0.05) and was more significant at 400 μg/mL concentrations (Figure 3C). The results suggested that TYHX increases the *I*_CaL_ current density of H/R-damaged SANCs.

### 3.4 Effects of TYHX on the gating mechanism of the *I*_CaL_

The above studies indicate that TYHX increases the amplitude and density of *I*_CaL_ current, but the mechanism of its regulation on the *I*_CaL_ gating mechanism is not understood. Therefore, we investigated the effects of TYHX on the steady-state activation (SSA) and the steady-state inactivation (SSI) curves as well as recovery from inactivation, activation kinetic, and deactivation kinetics.

#### 3.4.1 Effects of TYHX on the SSA and SSI curves of the *I*_CaL_

**FIGURE 4:**
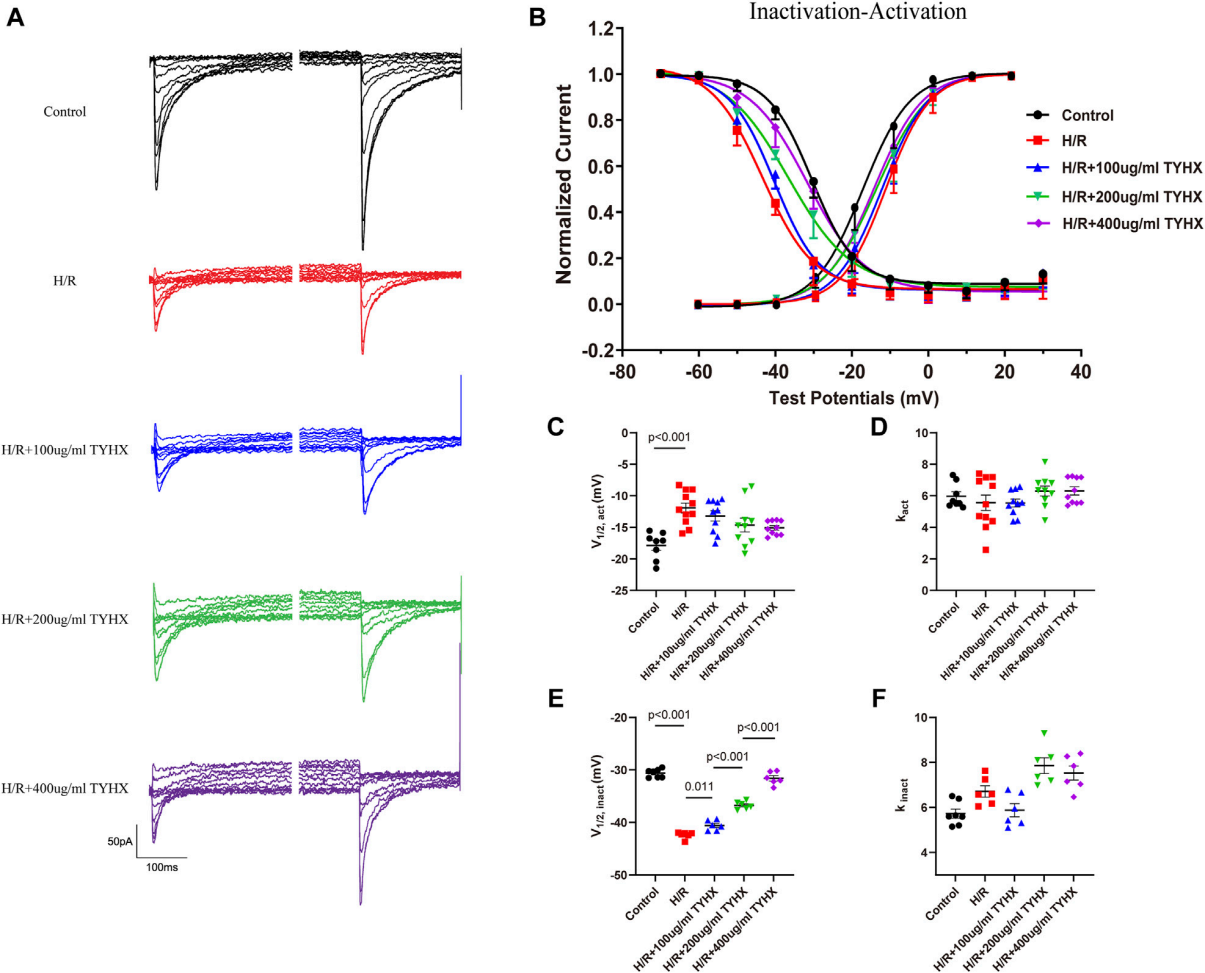
Effects of TYHX on the SSA and SSI Curves of the *I*_CaL_. **(A)** Pre-pulse and residual current of SSI curve of *I*_CaL_. **(B)** SSA and SSI curve of *I*_CaL_ (*n* = 10). **(C)** V_1/2, act_ of SSA curve (*n* = 10). **(D)** k_act_ of SSA curve (*n* = 10). **(E)** V_1/2, inact_ of SSI curve (*n* = 10). **(F)** k_inact_ of SSI curve (*n* = 10).

The SSA results showed (Figures 4B–D) that the SSA curve in the H/R group shifted significantly to the right (V_1/2, act_: H/R: 11.92 ± 0.79 vs. Ctrl: 17.86 ± 0.76 mV, n = 10, *p* < 0.01), and there was no significant change in k_act_ (*p* > 0.05). After TYHX intervention (Figures 4B–D), the SSA curve shifted to the left, but there was no significant difference in V_1/2, act_, and k_act_ between H/R group and different concentrations of the TYHX group (*p* > 0.05). It is suggested that TYHX has little impact on accelerating the steady-state activation of *I*_CaL_ in H/R-damaged SANC.

The SSI results (Figures 4A, B, E, F) showed that the SSI curve in the H/R group shifted significantly to the left (V_1/2, inact_: H/R: 42.41 ± 0.26 vs. Ctrl: 30.63 ± 0.30 mV, n = 10, *p* < 0.01); in TYHX intervention groups, the SSI curve shifted significantly to the right (V_1/2, inact_: 100 μg/mL: 40.58 ± 0.39 mV, 200 μg/mL: 36.76 ± 0.31 mV, 400 μg/mL: 31.60 ± 0.49 mV, n = 10, *p* < 0.05), with a statistical difference between groups (*p* < 0.01). There was no statistically difference in k_inact_ between the groups (*p* > 0.05).

The activation and inactivation curves of the calcium current revealed a voltage range between −40 mV and 0 mV where the window current was observed in control group (Figure 4B). After H/R damage, the window current became narrower. However, TYHX intervention resulted in a larger window current in H/R-damaged SANCs. These results suggest that TYHX may slow the steady-state inactivation of *I*_CaL_ channels and increase the window current, which may be one of the reasons why TYHX increases the amplitude and density of *I*_CaL_ currents.

#### 3.4.2 Effects of TYHX on the recovery from the inactivation of the *I*_CaL_

**FIGURE 5:**
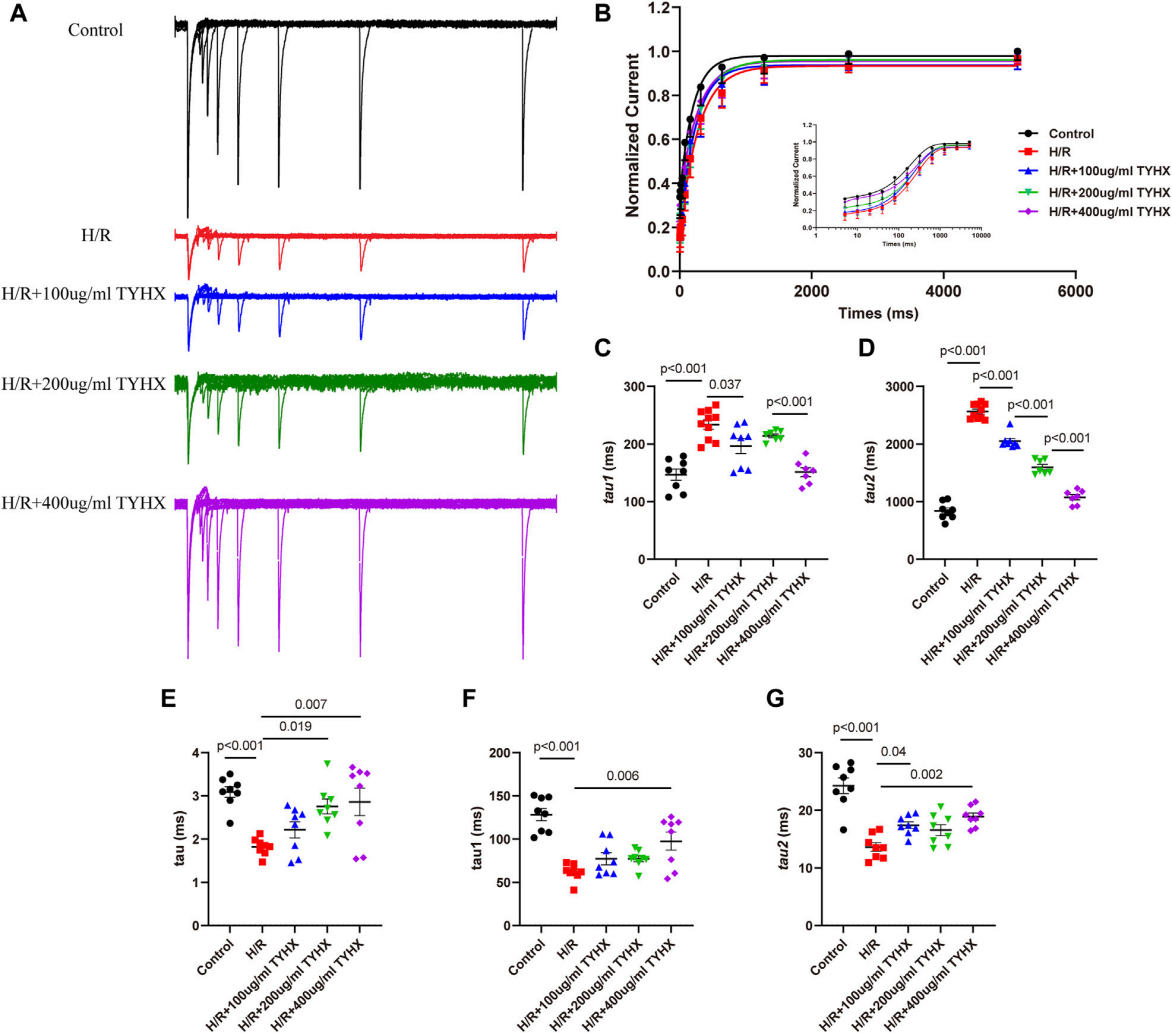
Effects of TYHX on the recovery from the inactivation, activation kinetics, and inactivation kinetics of the *I*_CaL_. **(A)** Recovery curve from the inactivation of *I*_CaL._ **(B)** Recovery from the inactivation of *I*_CaL_ (*n* = 7–10). **(C)** Fast recovery time constant τ_1_ (*n* = 7–10). **(D)** Slow recovery time constant τ_2_ (*n* = 7–10). **(E)** Activation time constant τ (n = 8). **(F)** Fast inactivation time constant τ_1_ (*n* = 8). **(G)** Slow inactivation time constant τ_2_ (*n* = 8).

Compared to the Control group, the H/R group exhibited slower recovery of *I*_CaL_ after inactivation (Figures 5A, B), with significantly prolonged fast recovery time constant τ_1_ and slow recovery time constant τ_2_ (τ_1_: H/R: 233.68 ± 8.11 vs. Ctrl: 147.00 ± 9.80 ms, n = 8–10, *p* < 0.01; τ_2_: H/R: 2564.30 ± 40.72 vs. Ctrl: 837.83 ± 52.45 ms, n = 8–10, *p* < 0.01) (Figures 5C, D). TYHX intervention shortened the recovery process of *I*_CaL_, with τ_1_ being shorter in response to 100 and 400 μg/mL concentrations of TYHX (100 μg/mL: 196.55 ± 12.95 ms, 400 μg/mL: 151.36 ± 7.83 ms, n = 7–8, *p* < 0.05), but no significant difference was observed in the 200 μg/mL group (*p* > 0.05). Additionally, τ_2_ was significantly shortened at different concentrations of TYHX (100 μg/mL: 2050.40 ± 44.86 ms, 200 μg/mL: 1598.43 ± 46.97 ms, 400 μg/mL: 1077.34 ± 46.51 ms, n = 7–8, *p* < 0.01). These results suggest that TYHX can accelerate the recovery process of *I*_CaL_ after H/R injury in sinus node cells, with 400 μg/mL concentration producing better effects.

#### 3.4.3 Effects of TYHX on the activation and inactivation kinetics of the *I*_CaL_

To explore the flow of ions per unit of time, we analyzed the mechanics of activation and deactivation. The results showed (Figure 5E) that activation time constant τ values significantly decreased in response to H/R (H/R: 1.82 ± 0.09 vs. Ctrl: 3.09 ± 0.12 ms, n = 8, *p* < 0.01). 200 and 400 μg/mL concentrations of TYHX increased τ values (200 μg/mL: 2.76 ± 0.18 ms, 400 μg/mL: 2.86 ± 0.33 ms, n = 8, *p* < 0.05).

When evaluating deactivation kinetics, it was found that both the fast inactivation time constant τ_1_ and slow deactivation time constant τ_2_ shortened in the H/R group (*p* < 0.01) (Figures 5F, G). In TYHX intervention groups, the fast inactivation time constant τ_1_ was prolonged by 400 μg/mL concentrations of TYHX (*p* < 0.01), while both 100 and 400 μg/mL concentrations prolonged the slow inactivation time constant τ_2_ (*p* < 0.05). The results suggested that TYHX can improve the activation and inactivation dynamics of H/R injury to a certain extent, and the effect of the 400 μg/mL-concentration group is more pronounced.

## 4 Discussion

### 4.1 Effects of TYHX promote the spontaneous AP and ignition phase

Through the above research, we found that changes in the ignition stage caused by hypoxia/reoxygenation injury are the key factor affecting the SANC’s autonomy. TYHX regulated the ignition phase to accelerate the transition from late DD to phase 0 depolarization and promote the autonomy of SANC, especially at 400 μg/mL concentrations.

The sinoatrial node cells are characterized by their automaticity, and pathological changes such as ischemia, fibrosis, and aging can lead to a decrease in their automaticity. Previous studies have shown that when the supply arteries of the sinoatrial node are obstructed, severe functional disorders may occur, ultimately leading to sudden death (Jing and Hu, 1997). Sinus dysfunction is a functional disorder of the sinoatrial node that manifests as a series of arrhythmias, including bradycardia, sinus arrest, and sick sinus syndrome. Ion channels on the cell membrane are the basis for the automaticity of SANCs. Various pathological injuries, including oxidative stress, apoptosis, and abnormal energy metabolism, can ultimately lead to abnormal changes in ion channels, affecting sinoatrial node automaticity.

The recent proposals of the “calcium clock” and “membrane clock” have deepened our understanding of the pacemaker mechanism of the sinoatrial node. This mechanism suggests that the calcium clock, which occurs in the sarcoplasmic reticulum, produces spontaneous and rhythmic local calcium release (LCR) through RyR and oscillates Ca^2+^ continuously and variably. It is coupled to the membrane clock, which produces current oscillations similar to limit-cycle oscillations (Weiss and Qu, 2020). The periodic opening and closing of ion channels on the cell membrane form the membrane clock, which also regulates the sarcoplasmic reticulum calcium concentration. The coupled clock is interdependent and regulates the automaticity of sinoatrial node cells. Other studies have also shown that the calcium clock and membrane clock are coupled by many mechanisms, including the coupling of sarcoplasmic reticulum LCR and NCX inward current, LTCC-mediated “resetting” and “replenishment” of the calcium clock (Maltsev and Lakatta, 2009), and so on.

Accumulating studies indicate that traditional Chinese medicine and its active ingredients can regulate the “calcium clock” and “membrane clock.” Herbal ingredients such as astragaloside iv, ginsenosides, and quercetin have been shown to inhibit calcium overload (Meng et al., 2005; Jing et al., 2016; Cui et al., 2023), thereby mitigating myocardial ischemic injury. Various Chinese medicine formulas also have been found to regulate currents like *I*_f_, *I*_CaL_, *I*_kur_, *I*_to_(Minoura et al., 2013; Liu et al., 2016; Wang et al., 2022). Previous research indicates that TYHX can inhibit ROS, preserve β-tubulin, and activate SIRT1, protecting mitochondrial function and subsequently alleviating hypoxic stress injury in SNCs (Chang et al., 2023). It seems that the regulation of these currents by traditional Chinese medicine is primarily achieved through antioxidant effects. Furthermore, our prior research has shown that TCM can enhance protein kinase A (PKA) activity in sinoatrial node cells, thereby reducing ischemia-reperfusion injury (Liu et al., 2016). The cyclic adenosine monophosphate (cAMP) -PKA signaling pathway regulates SANC function by modulating the coupling between calcium clock and the membrane clock. PKA is known to phosphorylate ion channels that pump Ca^2+^ out and release it from the sarcoplasmic reticulum, as well as to regulate enzymes involved in ATP production in mitochondria. Inhibition of PKA results in a biphasic decrease in action potential discharge rate (Mazgaoker and Yaniv, 2023). Therefore, TYHX may enhance the spontaneous pulsation of sinoatrial node cells by activating the cAMP-PKA signaling pathway.

Although significant progress has been made in the molecular and ionic mechanisms underlying the regulation of sinoatrial node automaticity, the complexity of controlling the overall pacemaker function remains to be fully explored, especially the coupling links of the “calcium clock” and “membrane clock.” Thus, we focus on how the Tongyang Huoxue Granule regulates the initiation and evolution of action potential in damaged sinoatrial node cells during the “ignition phase."

### 4.2 Effect of TYHX on *I*_NCX_

During the ignition phase, local calcium release in the SANCs triggers the inward sodium-calcium exchanger. At the same time, The NCX channel instantaneously couples the Ca^2+^ signal to the membrane clock, producing rapid effector mechanisms and activating L-type calcium channels to increase LCR activity (Yaniv et al., 2013). The process formed a feedback loop of “LCR-*I*_NCX_-*I*_CaL_-LCR” regulation and indicates that *I*_NCX_ and *I*_CaL_ provide functional connections between the “calcium clock” and “membrane clock."

To verify the above mechanism, we further explored the electrophysiological changes of *I*_NCX_ and *I*_CaL_ during the ignition phase. Our study showed that hypoxia/reoxygenation injury reduced the density of the inward *I*_NCX_ current, which may be the reason for delayed ignition in the damaged SANCs. The Tongyang Huoxue granules enhanced the voltage-dependent of the *I*_NCX_ in damaged SANCs. The result suggests that the regulation of Tongyang Huoxue granules may be achieved by increasing the forward transport of *I*_NCX_, promoting the transition from diastolic depolarization to phase 0.

*I*_NCX_ plays a crucial role in maintaining calcium homeostasis and SAN pacemaking in both physiological and pathological conditions. NCX has two working modes: forward and reverse modes. In the forward mode, the *I*_NCX_ channel pumps out 1 cytoplasmic Ca^2+^ and pumps in 3 Na^+^. When excess Na^+^ enters, or there is too much positive membrane potential, *I*_NCX_ switches to the reverse mode, pumping in 1 Ca^2+^ and pumping out 3 Na^+^. In the SAN, the activation of *I*_NCX_ in the forward mode could accelerate the occurrence of APs and reduce intracellular Ca^2+^ without the need for beta-adrenergic receptor agonists (Kohajda et al., 2020). When NCX is inactive, spontaneous beats weaken or disappear as a result of disrupted calcium homeostasis (Kohajda et al., 2020).

However, neither hypoxia/reoxygenation injury nor drug interventions significantly altered the outward current density of *I*_NCX_. This suggests that pathological changes such as hypoxia/reoxygenation and protective effects of natural drugs mainly affect the forward transport mode of *I*_NCX_ in SANCs. Besides, it may be due to the difficulty in separating outward currents of NCX in SANCs and the relatively small current density. Although some researchers have suggested that the peak voltage of the AP in the SANC is around 20 mV, which may activate the reverse mode of NCX, the exact role of NCX reverse mode in spontaneously beating cardiac myocytes has not been fully elucidated (Zhao et al., 2021). Furthermore, data modeling predicted only a small amount of reverse transport of *I*_NCX_ at the beginning of the action potential (Kohajda et al., 2020). Previous studies have demonstrated that the reverse mode of NCX plays a crucial role in activating RyR_2_ in mouse ventricular myocytes and directly regulates the action potential of heart failure cells (Armoundas et al., 2003). Therefore, with the availability of selective inhibitors of NCX transport mode in the future, further exploration can be made into the effect of Tongyang Huoxue Granules on NCX reverse transport mode.

### 4.3 Effect of TYHX on *I*_CaL_

Based on the above studies, we have confirmed that the forward mode of *I*_NCX_ is an important mechanism for triggering the AP when using Tongyang Huoxue Granules. However, an increase in the amplitude of *I*_NCX_ alone may result in competition for Ca^2+^ between NCX and SERCA, leading to a depletion of Ca^2+^ from the sarcoplasmic reticulum (Lyashkov et al., 2018). Without linked *I*_CaL_ calcium influx, this can inhibit the calcium clock and delay the ignition phase. Therefore, further studies are needed to investigate *I*_CaL_ and elucidate the specific mechanism of the ignition phase.

*I*_CaL_ is mediated by voltage-gated calcium ion channels and is widely distributed on the cell membrane of the sinoatrial node. In the coupled clock, *I*_CaL_ provides a Ca^2+^ influx during the upstroke of the AP while triggering RyR_2_-dependent calcium release from the sarcoplasmic reticulum, accelerating diastolic depolarization. Cav1.2 and Cav1.3 are the calcium channel proteins for *I*_CaL_ (Xu et al., 2003). An immunofluorescence study revealed that Cav1.3 co-localizes with the RyR within the sarcomere structure, whereas Cav1.2 is predominantly localized to the plasma membrane (Christel et al., 2012). The selective interaction between Cav1.3 and RyR-mediated Ca^2+^ release contributes to counteracting abnormal bradycardia (Christel et al., 2012). Clinical studies have shown that mutations in the encoding gene CACN1D of Cav1.3 lead to congenital sinoatrial node dysfunction (Baig et al., 2011). Similarly, animal experiments have found that knocking out Cav1.3 in mice results in sinus bradycardia and high-degree atrioventricular block, which is related to the destruction of cardiac automaticity and inhibition of late diastolic LCR (Baudot et al., 2020).

Pathological damage such as hypoxia and inflammation can induce fibrosis of the sinoatrial node tissue (Zhang et al., 2020), which could also reduce *I*_CaL_ by 50%. Previous studies have observed that hypoxia/reoxygenation injury leads to a decrease in Cav1.3 expression in SANCs and that TYHX protects damaged SANCs and restores Cav1.3 expression (Zhang et al., 2024). In this study, we also found that hypoxia/reoxygenation injury decreased *I*_CaL_ amplitude and density in SANCs, while TYHX increased current density and restored the I-V curve of *I*_CaL_ in damaged SANCs, partially reversing the *I*_CaL_ changes caused by hypoxia/reoxygenation injury.

The voltage-gated opening probability of L-type calcium channels depends on the cell membrane potential. When the channels open, the intracellular calcium concentration increases, triggering a series of physiological processes in SANCs. The open and closed states of the channels affect the amplitude and density of *I*_CaL_. To elucidate the mechanism of *I*_CaL_ current changes under injury and the effect of TYHX on the activation and inactivation of L-type calcium channels, we further studied the gating mechanism of *I*_CaL_.

Our study found that hypoxia/reoxygenation injury slowed the steady-state activation process, accelerated the steady-state inactivation process, and prolonged the recovery process after inactivation, leading to a decrease in the opening probability of L-type calcium channels. Furthermore, H/R also affected the activation and inactivation kinetics, shortening the channel opening time and reducing ion flow per unit time. This indicates that the decrease in the open probability and the shortening of the opening time of *I*_CaL_ channels are the main reasons for the decrease in *I*_CaL_ current density caused by H/R injury. However, in the TYHX groups, the steady-state inactivation process of damaged SANC *I*_CaL_ was slowed down, and the recovery after inactivation was accelerated. Additionally, we observed an increase in *I*_CaL_ opening probability. The results of activation and inactivation kinetics also showed that TYHX prolonged the opening time of *I*_CaL_, which increased the ion flow per unit time. Therefore, TYHX may increase the *I*_CaL_ current density by improving the gating mechanism of *I*_CaL_.

NCX and L-type calcium channels are both membrane currents and calcium-regulating proteins. Impairment or individual increase in their function can lead to calcium homeostasis imbalance. In the sinoatrial node, calcium current plays a major role during phase 0, while in the ventricle, selectively reducing the influx of Ca^2+^ during the later stage of the action potential effectively suppresses EAD (Angelini et al., 2021). Compared to single synthetic compounds, the antioxidant effects of TCM may improve the function of channels by reducing pathological damage, thus there is less likelihood of increasing ventricular arrhythmias. Therefore, TYHX may synchronize the increase in *I*_NCX_ and *I*_CaL_ current densities in damaged SANC through antioxidant effects. We have also demonstrated through this experiment that TYHX enhances calcium cycling in damaged SANC, which helps maintain calcium homeostasis and promotes the occurrence of the ignition phase.

### 4.4 Limitations and future directions

While our study demonstrates the significant electrophysiological effects of TYHX on SAN cells during H/R injury, several limitations must be acknowledged and addressed in future research. First, although we observed an increase in *I*_CaL_ current density with TYHX treatment, we did not distinguish between the contributions of the Cav1.2 and Cav1.3 isoforms of L-type calcium channels. This distinction is crucial as these isoforms play different roles in the SAN function, particularly in the Ca^2+^ clock mechanism. Future studies should utilize selective blockers to dissect the specific contributions of these isoforms. Additionally, while we focused on *I*_CaL_ and *I*_NCX_ currents, other critical ion channels involved in SAN automaticity, such as *I*_f_, T-type Ca^2+^ channels (CaV3.1), and NaV channels, were not comprehensively analyzed.

Another limitation is the potential variability in our cell preparations. Although we used a consistent methodology for isolating SAN cells, the inherent heterogeneity of the SAN tissue means that we may have recorded from non-pacemaker cells. Future studies should use more precise techniques to identify and isolate pacemaker cells, possibly incorporating genetic markers or advanced imaging methods.

Furthermore, our current experimental setup did not assess the chronic effects of TYHX treatment or its potential protective effects when administered as a pre-treatment before H/R injury. Evaluating the long-term impact of TYHX and its prophylactic capabilities could provide deeper insights into its therapeutic potential. We also recognize the need for *in vivo* studies to corroborate our *in vitro* findings. The complex *in vivo* environment, with its multiple interacting systems, could reveal additional effects and mechanisms not observed in isolated cell studies. Moreover, larger animal models with more anatomically and functionally similar SAN structures to humans would enhance the translational relevance of our findings.

## 5 Conclusion

**FIGURE 6:**
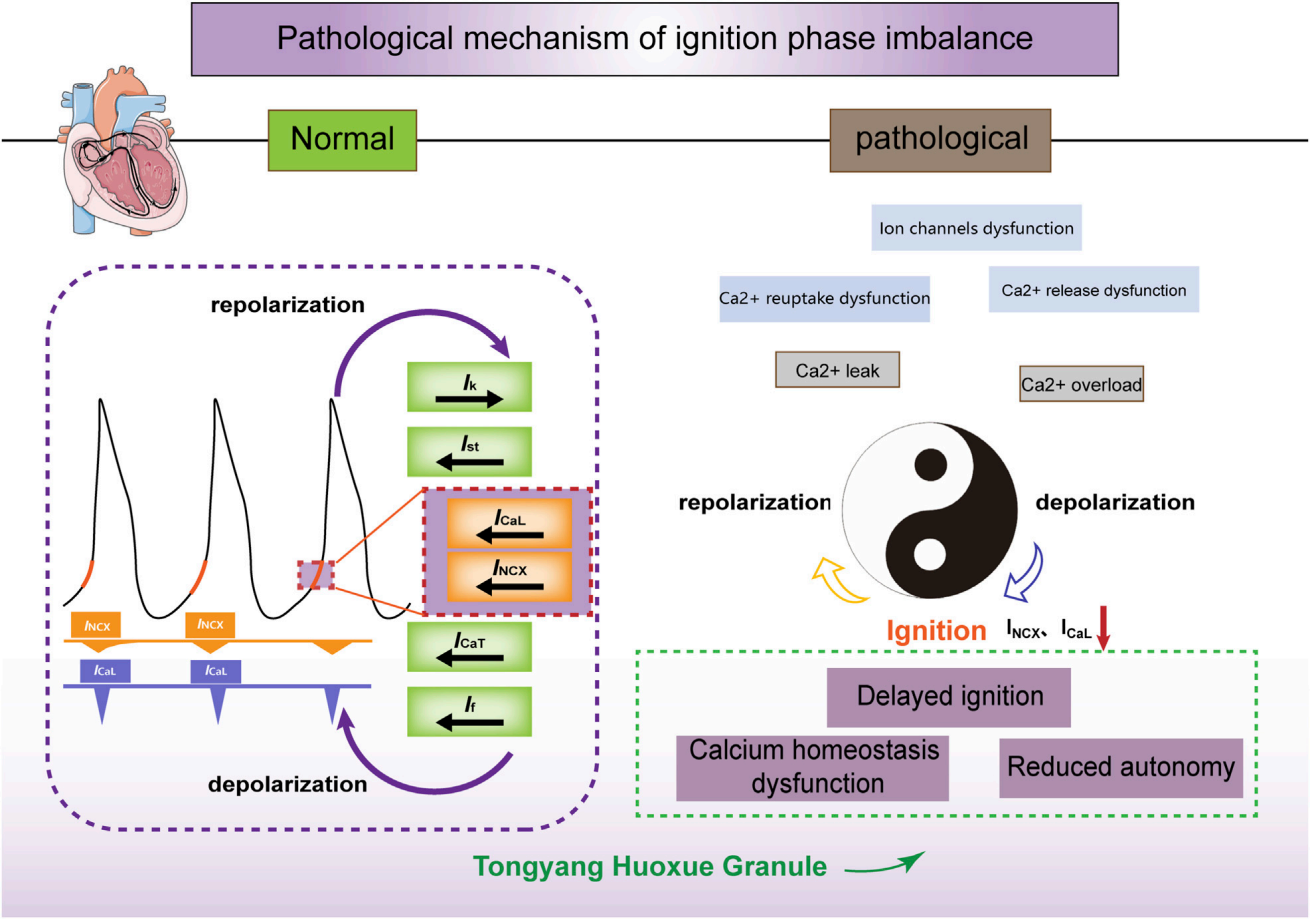
The ischemia/reperfusion-induced ignition phase imbalance and the effects of TYHX on *I*_NCX_ and *I*_CaL_. Figure legend: Under normal conditions, calcium release (calcium clock) within the sarcoplasmic reticulum is synchronized with the periodic opening and closing of ion channels and pumps on the cell membrane (membrane clock) to form a coupled clock system. *I*_NCX_ and *I*_CaL_ act on the initiation stage of action potentials, contributing to their spontaneous generation. In pathological conditions, hypoxia/reoxygenation leads to ion channel dysfunction and impaired Ca^2+^ reuptake and release, inhibiting the inward transport mode of *I*_NCX_ and *I*_CaL_. Consequently, this delay in the initiation phase and decrease in sinus node cell automaticity occur. Tongyang Huoxue Granule regulates calcium homeostasis and the initiation phase, thereby increasing the automaticity of sinus node cells.

The cycle of intracellular calcium recycling and release, membrane depolarization and repolarization, as well as the cycling of calcium influx through *I*_CaL_ and efflux through *I*_NCX_, is akin to the interplay of “Yin” and “Yang” (Figure 6). *I*_NCX_ and *I*_CaL_ maintain the coupling phase between the calcium and membrane clocks and drive the periodic rhythmicity of action potentials, which is a critical link in the sinoatrial node’s pacemaker mechanism. Through *in vitro* experiments, we have verified that TYHX accelerates the spontaneous beating of damaged sinoatrial node cells and regulates the “ignition phase” of the action potential. The mechanism mainly involves TYHX’s voltage-dependent promotion of the forward transport mode of *I*_NCX_ and the regulation of *I*_CaL_ through the gating mechanisms, which maintains calcium homeostasis.

## Data Availability

The raw data supporting the conclusion of this article will be made available by the authors, without undue reservation.
